# Scale-free dynamics and critical phenomena in cortical activity

**DOI:** 10.3389/fphys.2013.00079

**Published:** 2013-04-10

**Authors:** Tjeerd W. Boonstra, Biyu J. He, Andreas Daffertshofer

**Affiliations:** ^1^School of Psychiatry, University of New South WalesSydney, NSW, Australia; ^2^Black Dog InstituteSydney, NSW, Australia; ^3^Research Institute MOVE, VU University AmsterdamNetherlands; ^4^National Institute of Neurological Disorders and Stroke, National Institutes of HealthBethesda, MD, USA

The brain is composed of many interconnected neurons that form a complex system, from which thought, behavior, and creativity emerge. The organizing principles of complex networks can be investigated using approaches developed by modern complexity science (Albert and Barabási, 2002). Activity in many large networks including the brain has been shown to be scale-free, e.g., the spatiotemporal propagation of activity in multi-electrode local field potentials (LFP) obeys a power-law distribution—termed “neuronal avalanches” (Beggs and Plenz, 2003). Moreover, fluctuations in electrophysiological and neuroimaging signals reveal prevalent scale-free dynamics (Linkenkaer-Hansen et al., 2001; He, 2011). These studies have sparked resurgent interests in scale-free brain dynamics and raise the question whether the brain might be operating in a permanently critical state (Chialvo, 2004). These topics were discussed in a symposium at the 17th Annual Meeting of the Organization for Human Brain Mapping in Quebec City in 2011 and form the basis of this Research Topic in *Frontiers in Fractal Physiology*.

Notwithstanding recent advances, whether the brain is in a critical state remains unanswered. In a Socratic dialog, Beggs and Timme (2012) review recent literature providing evidence for this hypothesis. A central issue is whether power-law scaling can be convincingly shown in neural data and whether this is sufficient proof for criticality, as other processes may also produce power-law distributions. Solutions may include experimentally steering the system away from the critical point and investigating changes in scaling behavior. Despite increasing evidence supporting this hypothesis, the presence of critical states in the awake brain remains controversial.

Indeed, using high-density electrode array recordings of cortical activity in cats, monkey, and human subjects, Dehghani et al. (2012) showed that avalanche sizes derived from spiking data never revealed clear power-law scaling but scaled exponentially or displayed intermediate scaling. In contrast, simultaneously recorded LFPs did reveal evidence for power-law scaling in local peak sizes. Although their finding does not contradict those for criticality in neuronal slices (Beggs and Plenz, 2003) and anesthetized states (Hahn et al., 2010), it clearly argues against criticality as an encompassing principle for different brain systems.

The Research Topic revealed a broad range of recording techniques to assess scale-free dynamics. Monto (2012) investigated the dynamics of phase synchrony in magnetoencephalography (MEG). Nested synchrony was investigated by considering the phase coupling between faster oscillations in two distinct brain regions as a function of the phase of slow oscillations. Nested synchrony was sparsely but robustly present in MEG recordings of human brain activity. Although these data do not directly speak to the presence of scale-free dynamics, nested synchrony may be a candidate for organizing neuronal oscillations across time and spatial scales.

Hemodynamic responses are also a candidate modality for testing the presence of criticality in the brain. Ciuciu et al. (2012) examined the scaling properties of the temporal dynamics of fMRI signals. They employed multi-fractal analysis that quantifies a collection of scaling exponents rather than a single exponent. Scaling behavior during rest was compared to brain activity in an auditory detection task and revealed multi-fractal dynamics in functional networks as well as in artifacts. However, only functional components showed significant modulations of the multi-fractal attributes between rest and task.

Fraiman and Chialvo (2012) investigated the statistical properties of spatio-temporal dynamics in fMRI data. They considered three novel statistical features, which reveal the type of fluctuations generated by systems in a critical state. Their results showed that the variance of the fMRI signal remained constant across a wide range of observed cluster sizes, that this behavior originated from bursts of synchronized activity across regions, and that correlation length diverged so that clusters of different sizes exhibit the same collective dynamics.

The development of analytical techniques for the detection of scale invariance played a central role in many contributions. The review article by Hardstone et al. (2012) provides a comprehensive account of detrended fluctuation analysis (DFA), a method that has been widely used for analyzing scaling behavior. The review gives a detailed explanation of the underlying concepts and provides basic examples to clarify different types of scaling behavior. They then applied DFA to amplitude fluctuations in EEG data and provided an open-source software toolbox to encourage further use of this technique.

Several models have been investigated to understand mechanisms that may underlie scale-free dynamics. In a review article, de Arcangelis and Herrmann (2012) considered a new family of networks, the Apollonian networks (Andrade et al., 2005), which are scale-free and have small-world topology. Integrate-and-fire type neurons were connected as an Apollonian, a scale-free, or a fully connected network and synaptic strengths were modified according to a Hebbian learning rule. Once a percentage of connections were pruned, plastic adaption was stopped and the avalanche statistics and power spectral densities were assessed. Power-law behavior was observed for all models except for the fully connected networks showing supercritical behavior.

Aburn et al. (2012) investigated the scaling behavior of the Jansen-Rit model, a mean-field model that describes the average firing rate of interconnected neural populations. The bifurcation dynamics was studied as extrinsic input was delivered to different subpopulations. Long-range temporal correlations were assessed as a statistical indicator of linear instability and showed an increase of the autocorrelation length depended on the direction of the input fluctuations. Hence the detection of scale-free dynamics was dependent on the subpopulation that was stochastically perturbed, which has implications for applying these indicators to EEG recordings.

Finally, Van Orden et al. (2012) investigated the functional implications of critical brain dynamics by considering the body-brain relationship. Haken (1977) described that close to a phase transition, the components partition in two distinct groups in which slow macroscopic processes enslave fast microscopic processes. Hence the authors speculated that slow bodily processes may in fact control faster brain processes. They reviewed studies showing power-law scaling in human performance data and concluded that “metastable system can commit to a region of the state space of possibilities for action, without otherwise narrowing its options.”

This Research Topic reflects the heterogeneity of research on critical phenomena in cortical activity. The studies point out that the wide-ranging findings may not be reconciled with a single unifying theory. The theory of critical brain dynamics may be more like a searchlight theory (Popper, 1972), shedding new light on well-known things and creating new problems and observations. We should hence anticipate that the current theory may prove too coarse and requires further adjustments. The emphasis on network dynamics and the statistics of spatiotemporal fluctuations have provided key insights in brain organization and inspired new research directions. Unfortunately not all of us will be able to explore these new exciting possibilities. Guy van Orden passed away on 11 May 2012 while working on his manuscript for this Research Topic. His contributions will be truly missed.
